# Incidence angle and gray-to-white-matter ratio dependence of the focused-ultrasound induced blood-brain barrier opening in non-human primates

**DOI:** 10.1186/2050-5736-3-S1-P22

**Published:** 2015-06-30

**Authors:** Maria Eleni (Marilena) Karakatsani, Gesthimani Samiotaki, Matthew Downs, Elisa Konofagou

**Affiliations:** 1 Columbia University, New York, New York, United States

## Background/introduction

FUS coupled with the systemic administration of microbubbles has been proved to open the Blood-Brain Barrier (BBB) locally, transiently and non-invasively in non-human primates (NHP). However, the complexity of the NHP brain constitutes an obstacle in relating the volume size of the induced opening (VBBB) to the geometric aspects of the method as well as the physiologic characteristics of the targeted areas. The objective of the current study is to quantify the correlation between the VBBB, the FUS pressure and the incidence angle. Additionally, the dependence of the opening shift on the gray-to-white-matter ratio at the targeted area is studied.

## Methods

Five (n=5) NHP, i.e., four macaques of the mulatta and one fascicularis, were sonicated in two brain structures, the caudate (Cau) and putamen (Pu) using FUS (F0: 500kHz; PRF: 2Hz; duration 120s; PNP 300-600 kPa) while being intravenously administered with monodisperse (4-5 micron in diameter) microbubbles. The NHPs were scanned in a 3T MR scanner (Philips, USA) acquiring 3D T1 weighted (T1w) pre- and post-contrast images approximately 24 hours after BBB opening to allow for behavioral assessment in a separate study. The estimation of the incidence angle and the center of the targeted area was were by projecting the ultrasound beam propagation and the focal region onto the BBB opening site detected on post-contrast T1w images. To estimate the shift, an automated intensity-based algorithm was designed that identified the centroid of the actual opening and the VBBB. Finally, the opening was overlayed onto the brain region on a thresholded T1w image (figure) to quantify the percentage of gray matter (GM) and white matter (WM) affected from FUS.

**Figure 1:**
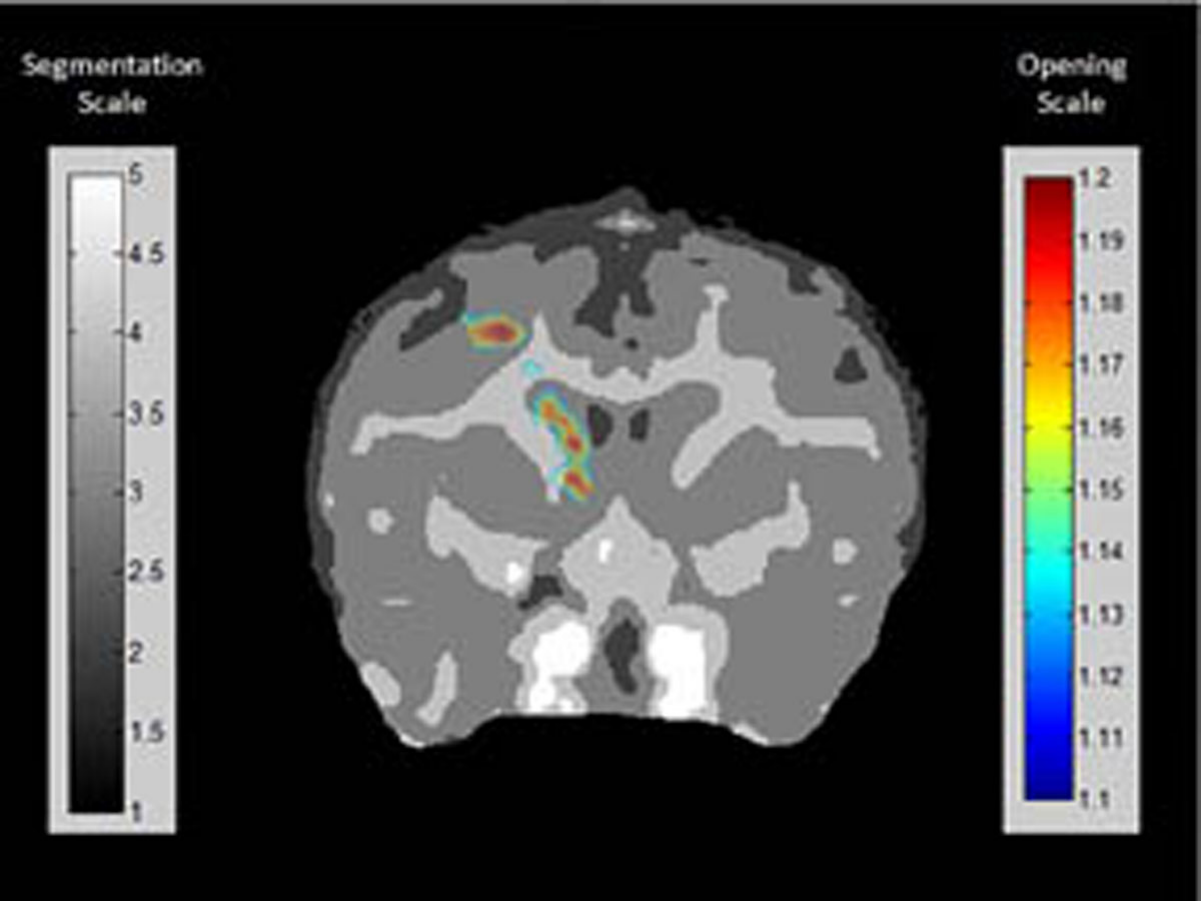
Overlay of the BBB opening onto a thresholded T1w image.

## Results and conclusions

VBBB increased from 106.79±50 mm3 to 305.7±50 mm3 at an incidence angle range of 73.67 ± 0.19° to 89.83 ± 0.19° at 300kPa, respectively. Similar increase was obtained at other pressures suggesting a linear correlation between the three aforementioned components. The squared correlation coefficient (R2) varied from 0.71 to 0.97 when studying each NHP separately. The opening in the GM accounted for an average of 88.5±8.9% of the opening cases when targeting the Pu, while 78.3±6.6% occurred in the GM when focusing on the Cau. In conclusion, the VBBB was found to strongly depend on both the incidence angle and the pressure applied. It was also concluded that the shift in the BBB opening region from the targeted one depends on the gray-to-white-matter ratio present in the focal region.

